# Healing Rates in a Multicenter Assessment of a Sterile, Room Temperature, Acellular Dermal Matrix Versus Conventional Care Wound Management and an Active Comparator in the Treatment of Full-Thickness Diabetic Foot Ulcers

**Published:** 2016-02-04

**Authors:** Jodi Walters, Shawn Cazzell, Hau Pham, Dean Vayser, Alexander Reyzelman

**Affiliations:** ^a^Southern Arizona VA Health Care System, Tucson; ^b^Limb Preservation Platform, Valley Vascular Surgical Associates, Fresno, Calif; ^c^Boston University School of Medicine, Boston Medical Center, Boston, Mass; ^d^ILD Research Center, San Diego, Calif; ^e^California School of Podiatric Medicine at Samuel Merritt University, Oakland; ^f^UCSF Center for Limb Preservation, San Francisco, Calif

**Keywords:** diabetic foot ulcer, wound, acellular dermal matrix, allograft, DermACELL

## Abstract

**Objective:** The purpose of this 16-week, multicenter, randomized, controlled trial was to assess the healed ulcer rate of a human acellular dermal matrix, DermACELL, compared with conventional care and a second acellular dermal matrix, Graftjacket, in the treatment of full-thickness diabetic foot ulcers. **Methods:** One hundred sixty-eight patients were randomized into DermACELL, conventional care, and Graftjacket treatment arms in a 2:2:1 ratio. Patients in the acellular dermal matrix groups received either 1 or 2 applications of the graft at the discretion of the investigator. Weekly follow-up visits were conducted until the ulcer healed or the endpoint was reached. **Results:** At 16 weeks, the DermACELL arm had a significantly higher proportion of completely healed ulcers than the conventional care arm (67.9% vs 48.1%; *P* = .0385) and a nonsignificantly higher proportion than the Graftjacket arm (67.9% vs 47.8%; *P* = .1149). The DermACELL arm also exhibited a greater average percent reduction in wound area than the conventional care arm (91.4% vs 80.3%; *P* = .0791) and the Graftjacket arm (91.4% vs 73.5%; *P* = .0762). The proportion of severe adverse events and the proportion of overall early withdrawals were similar among the 3 groups based on relative population size (*P* ≥ .05). **Conclusions:** The results presented here indicate that DermACELL is an appropriate clinical option in the treatment of diabetic foot ulcers, with significant increases in healing rates and rate of percentage wound closure as compared with conventional care options.

An estimated 29.1 million people currently suffer from diabetes.^1^ The most common complication among diabetic patients is neuropathy, a condition involving poor sensation in the extremities.^2^ Around 8% of newly diagnosed diabetic patients and more than 50% of patients with chronic diabetes will develop neuropathy,^3^ which is a contributory cause of diabetic foot ulcers (DFUs). It is estimated that the lifetime risk for developing a foot ulcer among the diabetic population is 25%.^4^ DFUs can severely impact patients’ quality of life and health. Patients with DFUs report significantly worse quality of life, including significantly lower levels of physical functioning, social functioning, physical role, and health experience than patients without DFUs.^5^ These difficult-to-treat ulcers can also lead to serious complications such as amputation or death, with the 5-year mortality rate of patients with DFUs at 40%.^6^ In addition to affecting the quality of life and health of patients, DFUs are expensive to treat. The cost of treating a single diabetic patient with a DFU in the United States averaged $31,419 over 1 year, more than twice the expense of a diabetic patient without a DFU.^7^

The current standard conventional care for DFUs includes debridement, off-loading of the wound, proper dressing of the wound, and infection control, if necessary.^8^ Standard care has a wide range of 12- and 16-week healing rates, with rates reported in the literature between 21.3% and 46.2%.^9^^-^12 An alternative treatment of DFUs is a human acellular dermal matrix (ADM), which can provide a scaffold for tissue growth. There are few large-scale studies evaluating ADM use in DFUs, but 12-week healing rates are reported as high as 69.6%.^11^^,^^13^ A particular ADM, DermACELL and referred to hereafter as D-ADM, has shown success in early case series,^14^^,^^15^ which prompted this larger trial. D-ADM is prepared using a unique decellularization process,^16^ resulting in a material with thorough DNA removal, retention of biomechanical strength, and provided fully hydrated at room temperature. The product is also terminally sterilized to a sterility assurance level of 1 × 10^−6^, consistent with medical device regulations, although classified as a human tissue.^16^ The purpose of this study was to compare the healing rate and other healing metrics of D-ADM with conventional care and an active comparator, Graftjacket (hereafter referred to as GJ-ADM).

## METHODS AND MATERIALS

### Design and objectives

This study was a multicenter, randomized, controlled, open-label trial designed to evaluate the safety and efficacy of D-ADM compared with conventional wound care management and GJ-ADM in patients with chronic DFUs (Clinical trial registration number NCT01970163, http://ClinicalTrials.gov). The study design, methods, and informed consent were reviewed and approved by a central institutional review board (IRB), Western International Review Board, as well as local IRBs. There were 11 implanting surgeons from 10 medical centers in 8 states. Patients were randomly assigned to the D-ADM, conventional care treatment, and GJ-ADM arms in a 2:2:1 ratio. The ADM arms contained patients who received 1 or 2 ADM applications, a second application being applied at the discretion of the study site principal investigator. For this analysis, there were 53 patients in the D-ADM group, 56 patients in the conventional care group, and 23 patients in the GJ-ADM group. The primary endpoint of this study analysis was assessment of complete reepithelialization with no drainage or dressing requirements up to 16 weeks. In further stringency, an assessment of wound closure required confirmation at 2 consecutive study visits performed 2 weeks apart. The healing rate of wounds at 16 weeks and the percentage of reduction in wound size from baseline up to 16 weeks were also analyzed. The primary study hypothesis was that D-ADM patients would exhibit a higher proportion of healed wounds at 12 weeks than those treated with conventional care.

### Assessment methods

Wounds were evaluated on a weekly basis until wound closure was observed or 16 weekly follow-up visits passed. Wound closure was defined as 100% reepithelialization of the wound without drainage. A second visit took place 2 weeks after initial wound closure observation to confirm complete wound closure in accordance with Food and Drug Administration (FDA) guidance and Agency for Healthcare Research and Quality (AHRQ) recommendations. Surface area of the wound and depth of the ulcer were measured and recorded at each visit. Measurements of the wound area were taken and recorded using Silhouette Advanced Wound Assessment and Management System (Aranz Medical, Merivale, Christchurch, New Zealand). Information was also collected on adverse events and concomitant medications throughout the study.

### Patient population

Patient demographics, shown in Table 1, were collected during each screening visit to determine a patient's eligibility for the study. All patients whose ulcers healed or who reached at least 12 weeks actively enrolled in the study without early withdrawal were included in the analysis. To be included in the study, patients must have given voluntary consent and met all inclusion criteria while avoiding all exclusion criteria. Inclusion criteria included, but were not limited to, the patient having a single, full-thickness target DFU with a Wagner ulcer classification grade of 1 or 2, a wound area of 1 cm^2^ or greater and less than 25 cm^2^, and a wound depth of 9 mm or less. Other inclusion criteria included the patient having had adequate circulation to the affected area, defined as having at least one of the following criteria within the past 60 days: transcutaneous oxygen measurement at the dorsum of the foot 30 mm Hg or more; ankle-brachial index ranging from 0.8 to 1.2; or at least biphasic Doppler arterial waveforms at the dorsalis pedis and posterior tibial arteries. Exclusion criteria included, but were not limited to, circulating hemoglobin A_1c_ exceeding 12% within 90 days of the screening visit, serum creatinine concentrations of 3.0 mg/dL or greater within 30 days before screening, having had wound treatments involving biomedical or topical growth factors within 30 days before screening, having undergone a revascularization procedure aimed at increasing blood flow in the target limb, or receiving a living skin equivalent within 4 weeks before screening.

### Surgical procedure

**Figure 1:**
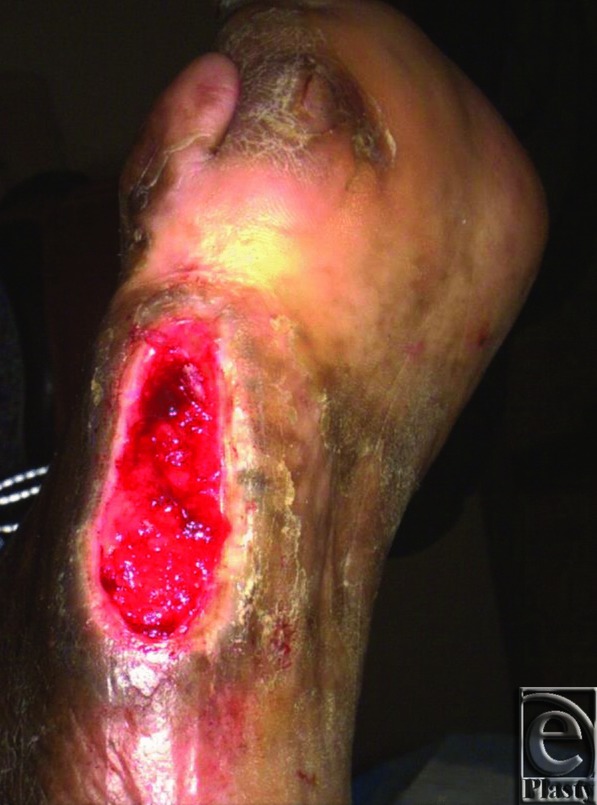
Preoperative diabetic foot ulcer at baseline with an area of 6.4 cm^2^ after debridement.

**Figure 2:**
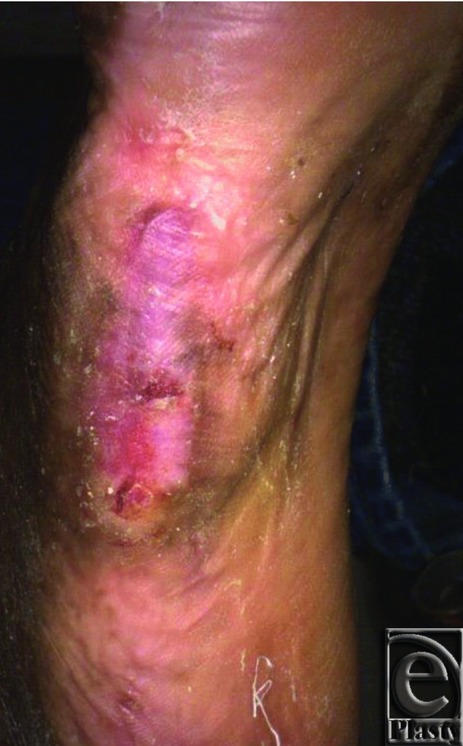
Wound was completely closed at 12 weeks following treatment with a single application of D-ADM.

At baseline, all wounds were debrided to remove necrotic tissue. Before and after debridement, but before treatment, wound size was recorded using the Silhouette system. Meshed, 4 × 4 cm (thickness range, 0.5 to 1.0 mm) D-ADM (DermACELL; LifeNet Health, Virginia Beach, Va) or meshed, 4 × 4 cm (thickness range, 0.38 to 1.02 mm) GJ-ADM (Graftjacket; Wright Medical Technology, Memphis, Tenn) was applied to patients in the D-ADM and GJ-ADM arms and covered with an appropriate nonadherent dressing. Depending on the state of the wound (dry or moist), different types of nonadherent dressings were utilized as the primary dressing (Table 2). If the wound was dry, a dressing was applied that tended to donate moisture to the wound, such as an oil emulsion dressing. Hydrogels were used if the wound was in need of moisture. For a wound that was more moist, an absorptive dressing to help reduce potential for maceration was suggested. A secondary dressing was allowed to add either loft or cushion. Investigators were given the option of bolstering the ADM with gauze pads before covering with gauze. If determined medically necessary by the investigator, a second ADM application was allowed to be administered no fewer than 3 weeks but no longer than 12 weeks (weeks 3–12) after the first application. Wounds in the conventional care arm underwent advanced moist wound therapy consisting of alginates, foams, or hydrogels, at the discretion of the study site principal investigator, and were then covered with the appropriate gauze. Each investigator was supplied with a listing of approved dressings to standardize the wound care across all treatment arms. Investigators were encouraged to follow their clinic wound care policies regarding use of compression wraps. Per the protocol, the dressings in all groups were only changed by the study team at each weekly follow-up visit. Negative pressure wound therapy and hyperbaric treatments were allowed at the investigators’ discretion during the trial. However, negative pressure wound therapy was used as an ancillary treatment at the baseline visit for only 2 subjects, one in the conventional care arm and one in the GJ-ADM arm, and hyperbaric treatments were not used at all during the study. Off-loading using a removable cast walker, diabetic shoe, surgical shoe, walker cast, or a total contact cast was required for all treatment arms unless the investigator deemed it was not appropriate, such as in those cases that the subject was wheelchair bound or the wound was on the dorsal surface of the foot. Although either removable or nonremovable off-loading methods were allowed, 92% of all patients used some sort of removable method, with 74% of those using removable boots. The small numbers using other methods did not allow for any meaningful numerical comparisons. Weekly follow-up visits occurred until full wound closure was observed (100% reepithelialization) (Figs 1 and 2) or the 16th week follow-up visit was reached. If wound closure was observed, a second visit occurred 2 weeks later to confirm wound closure and was considered the termination visit if the wound was still closed. Otherwise, the patient continued weekly follow-up visits until wound closure was observed or 16 weeks was reached. Follow-up visits occurred at 4, 8, and 12 weeks following final confirmation of complete wound closure.

## RESULTS

**Figure 3:**
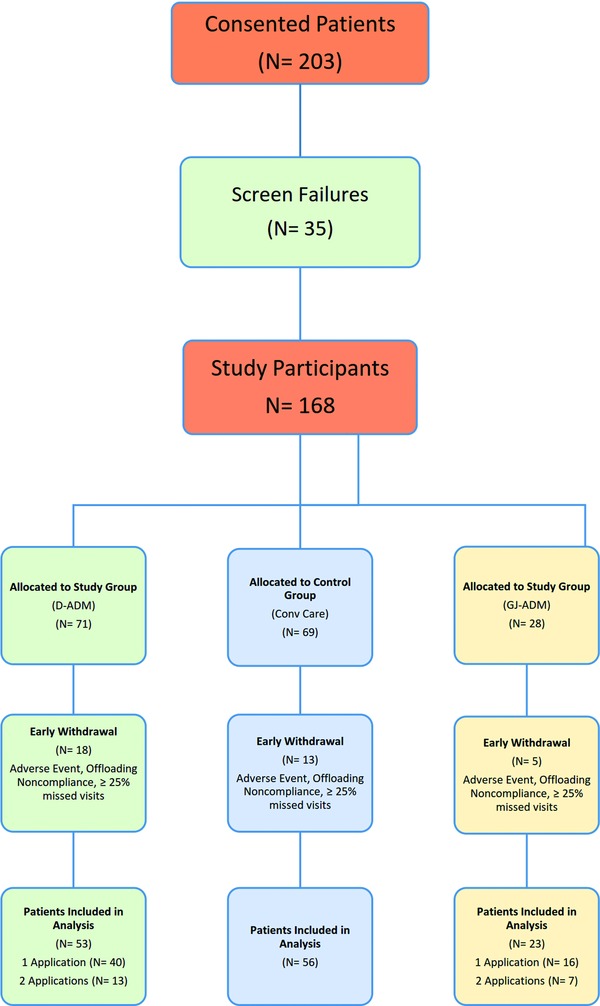
Flowchart depicting patient population over the course of the study.

The progression of patients through the study, from enrollment to completion, is shown in Figure 3. After screening, the remaining 168 eligible participants were randomly allocated to treatment arms. Throughout the course of the study, 18 patients in the D-ADM arm, 13 patients in the conventional care arm, and 5 patients in the GJ-ADM arm withdrew early for either an adverse event or significant noncompliance. The proportion of severe adverse events (SAEs) and the proportion of overall early withdrawals were similar among the 3 groups based on relative population size (*P* ≥ .05). The baseline ulcer size was also similar for each arm, with no observed statistical differences (Table 3). Fifty-three D-ADM patients, 56 conventional care patients, and 23 GJ-ADM patients whose ulcer healed or reached the 12-week follow-up visit and were included in this interim analysis. At week 13, one conventional care patient withdrew consent and a second conventional care patient was withdrawn by the investigator for a lack of wound healing and increased wound size. As they had completed the endpoint at 12 weeks, the data for both of these patients were included through week 13. Hypothesis testing was performed using *t* tests or χ^2^ tests at a 2-sided α of .05. Sample sizes were chosen to give a power of at least 0.8 for significant differences between the D-ADM and conventional care arms.

**Figure 4:**
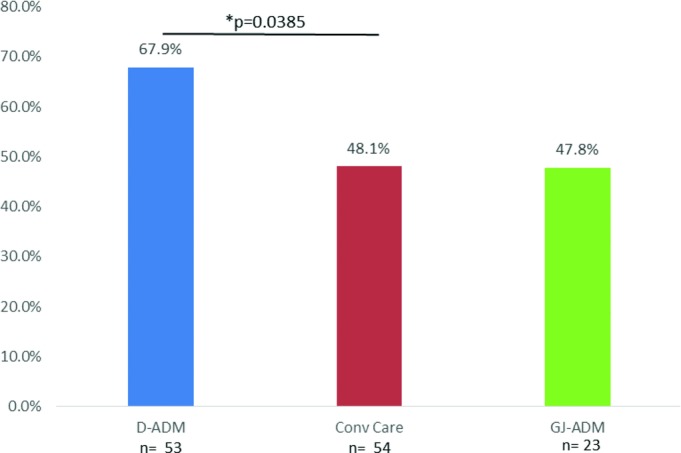
Percentage of healed wounds at 16-week follow-up. A statistically significant difference was seen for combined D-ADM versus Conv Care (*P* = .0385). No statistically significant differences were noted between D-ADM and GJ-ADM, nor between GJ-ADM and Conv Care. Conv Care indicates conventional care.

**Figure 5:**
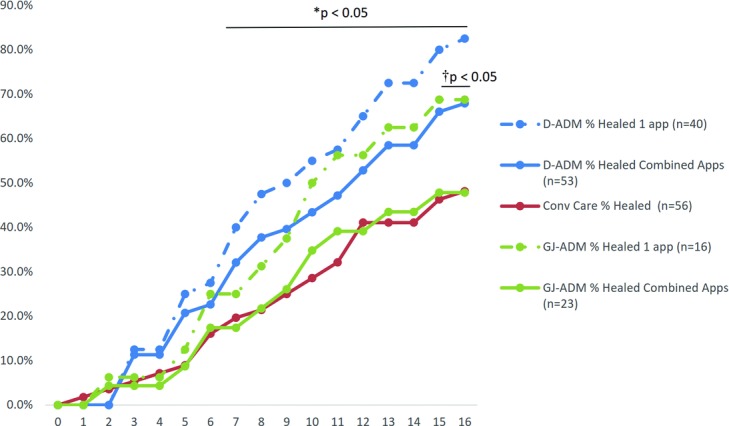
Percentage of healed wounds through 16 weeks. No statistically significant differences were noted between D-ADM and GJ-ADM, nor between GJ-ADM and Conv Care at any time point. *Statistically significant difference between 1-application D-ADM and Conv Care for weeks 7 to 16. †Statistically significant difference between combined application D-ADM and Conv Care for weeks 15 and 16. Conv Care indicates conventional care; app(s), application(s).

**Figure 6:**
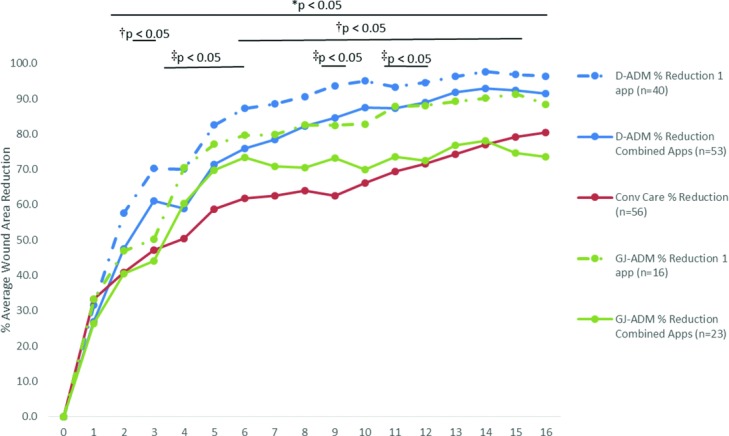
Percent average wound area reduction through 16 weeks. *Statistically significant difference between 1-application D-ADM and Conv Care for weeks 2 to 16. ^†^Statistically significant difference between combined application D-ADM and Conv Care for weeks 3 and 6–15. ^‡^Statistically significant difference between single-application GJ-ADM and Conv Care for weeks 4–6, 9, and 11–12. Conv Care indicates conventional care; app(s), application(s).

D-ADM demonstrated a greater wound closure rate and wound area reduction rate over both GJ-ADM and conventional care at 12 and 16 weeks (Table 4). The combined application, whether a single or second application was used, D-ADM-treated wounds exhibited a significantly higher full closure rate (*P* < .05) than conventional care at week 16 (Fig 4). ADM receiving patients were also separately evaluated by whether they received either 1 or 2 applications and compared with conventional care. ADM patients who received second applications were evaluated from the time of the first application at baseline; the baseline week was not changed to the week of the second application. For both ADM types, patients who had only received 1 application of ADM exhibited higher healing rates than the total application groups. Both 1-application and combined application D-ADM groups displayed significantly higher healed wound rates than conventional care at multiple time points throughout the 16-week follow-up (Fig 5). The difference in percent average wound area reduction was significant at weeks 3 and 6–15 for D-ADM versus conventional care (*P* < .05) (Fig 6). GJ-ADM displayed a similar wound reduction rate over conventional care at 12 weeks (72.5% vs 71.6%) but dropped to approximately 7% lower than conventional care's healed rate by 16 weeks (73.5% vs 80.3%). Among ulcers that did not heal by 16 weeks, D-ADM exhibited greater percent mean wound size reduction than conventional care and GJ-ADM (Table 5).

For 1-application groups, at the primary endpoint of 12 weeks, 1-application-only D-ADM patients exhibited significantly higher wound closure rates than conventional care patients (65.0% vs 41.1%; *P* = .0203) and a higher closure rate than patients who had received only 1-application GJ-ADM (65.0% vs 56.3%; *P* = .5624) (Fig 5). The greater healing rates for single-application D-ADM continued at 16 weeks, with D-ADM exhibiting a significantly higher rate than conventional care (82.5% vs 48.1%; *P* = .0003) and with a strong but nonsignificant increase over GJ-ADM (82.5% vs 68.8%; *P* = .3163).

## DISCUSSION

The results presented here demonstrate that D-ADM, a sterile, room temperature, decellularized dermal matrix, can be used safely and successfully to heal full-thickness DFUs. At the primary endpoint of 12 weeks, 1-application D-ADM had a significantly greater healed rate than conventional care (65.0% vs 41.1%; *P* = .0203) and a higher, though nonsignificant, healed rate than 1-application GJ-ADM (65.0% vs 56.3%; *P* = .5624). Although viewing single-application patients at 12 weeks is useful for comparison with earlier studies in the literature,^11^ evaluating the results of all application patients at 16 weeks is more consistent with recent literature^12^ and more realistically includes all treated patients. As reported here, at 16 weeks, combined analysis of all D-ADM patients demonstrated a statistically significant higher healed rate than conventional care (67.9% vs 48.1%; *P* = .0385) and a substantially higher rate than GJ-ADM that trended toward significance (67.9% vs 47.8%; *P* = .1149). The similar average baseline ulcer areas among the 3 treatment arms enabled a fair comparison. Both the D-ADM and GJ-ADM arms experienced fewer cases of SAEs than conventional care (4, 4, and 6 SAEs, respectively), which further supported the safety of D-ADM and GJ-ADM.

It is important to note that the study reported here uses a more rigorous healed ulcer criteria than other reports and as outlined in the FDA guidance on skin substitutes and the 2011 report from the AHRQ on the design of products to assist with wound healing.^17^^,^^18^ This study required that an ulcer must demonstrate complete healing on 2 consecutive visits to be considered healed rather than being considered healed at the first instance of wound closure. The more stringent healed ulcer criteria should be taken into account when comparing the healing rates of this study with others that have been published, especially those before 2011. Several ulcers in this study had 100% wound size reduction at a given week but were not considered healed because of these criteria.

The difference in healing criteria becomes more evident when contrasted with the Reyzelman et al^11^ study, where not a single nonhealing ulcer had a wound size reduction of 100%, yet significant healing rates were reported. Reyzelman et al found 12-week DFU healing rates of 69.6% for GJ-ADM and 46.2% for conventional care. While this ADM healing rate is higher than either of the ADM groups presented here, Reyzelman et al only included 1-application GJ-ADM patients in their results. With this taken into account, the single-application D-ADM group in the study reported here had a similar healing rate (65.0%), although the single-application GJ-ADM did not compare as well (56.3%). It is more difficult to make a direct comparison with the Winters et al^13^ study, as that GJ-ADM study lacked a control and did not evaluate healing at a certain week but rather allowed patients’ ulcer to heal without a study endpoint. However, D-ADM–treated ulcers that healed by 12 weeks in this study exhibited a much lower average time to healing of 7.0 weeks compared with the 13.8 weeks for GJ-ADM reported by Winters et al.

The results presented here for D-ADM compare favorably with those recently reported for Integra Dermal Regeneration Template (IDRT) for 16-week DFU healing rates.^12^ IDRT, an acellular bilayer matrix, was compared with conventional care for the treatment of DFUs in a 307-patient randomized controlled trial with a 16-week follow-up. The 16-week healing rate for combined D-ADM was 68% versus 51% for IDRT. It should also be noted that of the 53 D-ADM patients, 40 patients received 1 application and 13 patients received 2 applications of D-ADM. In contrast, IDRT patients received as many as 15 applications. To ensure an accurate comparison, baseline ulcer measurements were reviewed (Table 3) and the mean baseline ulcer size was approximately equivalent for D-ADM, IDRT, and GJ-ADM from the 2009 Reyzelman et al^11^ study. When comparing studies, it should also be taken into account that all ulcers in this reported study were full-thickness wounds and the majority of the ulcers were Wagner grade 2, which can be more difficult to heal.

In this study, both complete wound healing and wound size reduction were assessed throughout the patient treatment period. A greater reduction in wound size may provide several benefits including early prediction of successful healing, reversal of ulcer gradation using the Wagner scale, and improvement in performance on foot pressure tests.^19^^-^21 D-ADM–treated ulcers demonstrated a greater reduction in wound size than both the conventional care and GJ-ADM arms at 12 weeks and 16 weeks, respectively, for all wounds (healed and nonhealed) and among nonhealing ulcers alone (Tables 4 and 5). The benefits of ulcer size reduction on wound care and the improved capability of D-ADM in doing so indicate a potentially important area for further study.

DFUs have a high prevalence of recurrence, given their neuropathic nature and frequent location on plantar surfaces or other weight-bearing surfaces subject to increased pressure.^22^ Patients may walk abnormally to alleviate pressure on the ulcer, but this increases the likelihood of a recurrence on a nearby site.^23^ Rapid healing and reduction in size of the initial ulcer should be a priority goal to prevent these recurrences. In addition, the greater healing rate for 1-application D-ADM, noted here, continued at 16 weeks, which strongly indicated that the patients whose ulcers were not healed or needed second applications were most likely the patients who either were noncompliant, otherwise nonhealing, or had wounds in locations that would not likely heal with conventional care. Higher healed ulcer rates are critical to reducing the escalating cost of treating DFUs faced by both patients and the health care system.^7^ Amputation and hospitalization expenses averaged $18,084 for a minor procedure and $13,258 per stay,^24^ respectively, making early and effective treatment important to avoid spiraling costs. The greater healing rate of D-ADM, along with its long shelf life at ambient temperature and easy application process, makes this treatment option a potential benefit for patients and providers.

A common limitation in wound-healing studies is the absence of a blinded analysis of wound healing. While this analysis likewise relied on the disposition by the respective principal investigators of whether a wound was healed, we also sought the opinion of a blinded third-party adjudicator. At the 12-week primary endpoint, more than 87% agreement in assessment of complete healing was obtained between the blinded adjudicator and the principal investigator. The adjudicator judged 2 additional patients as completely healed for the D-ADM arm, 1 additional patient completely healed for the GJ-ADM arm, and reported no difference in healing for conventional care. The similar healed wound proportions determined by blind review support a lack of bias by the study investigators in deciding healed wound designations.

One limitation in this study's protocol was it allowed investigators to have discretion when using second applications of ADM instead of following firm guidelines. The lack of uniformity across different centers may have lowered the healing rates for both D-ADM and GJ-ADM in some instances. There may have been wounds that may have healed faster had a second application been applied. Likewise, some wounds could have gone to healing without a second application since it appears that a few patients received this second treatment before the wound healing had actually arrested. Future research could provide guidance on whether and when a second application of ADM would be warranted.

## CONCLUSION

The results presented here indicate that D-ADM (DermACELL) is an appropriate clinical option in the treatment of DFUs with significant increases in healing rates and rate of percentage of wound closure as compared with conventional care options.

## Figures and Tables

**Table 1:** Comparison of demographic variables between treatment groups

	Conv Care (*N* = 56)	D-ADM (*N* = 53)	GJ-ADM (*N* = 23)
Age, y			
Mean	57.1	58.0	58.7
Median	56.0	57.0	61.0
SD	10.9	13.1	10.4
Range	33–85	24–85	34–80
BMI			
Mean	32.9	31.2	31.7
Median	31.5	31.4	32.2
SD	6.8	5.7	5.3
Range	18.6–50.2	19.9–44.6	23.4–44.2
Diabetes type*			
Type 1	1 (1.8%)	1 (1.9%)	2 (8.7%)
Type 2	55 (98.2%)	51 (96.2%)	21 (91.3%)

*One patient in the D-ADM arm was considered prediabetic. Conv Care indicates conventional care; BMI, body mass index.

**Table 2:** Approved dressings for patient care

Oil emulsion	Hydrogels	Foams	Gauze	Alginates
Integrity	Derma-Gel	Dermafoam	Kendall 4 x 4	Gentell
Invacare	Elasto-Gel	Optifoam	Curity Fluffs	Silvercel
Curad	Flexigel	Covidien	J & J Gauze	Tagaderm High Gelling
Kendall Curity	Restore	Deroyal Polyderm	Kerlix Bandage Rolls	Tagaderm High Integrity
Shur-Conform	Carrasyn	Allevyn Foam	Kerlix Lite Bandage Rolls	Maxorb Extra
Adaptic	Vigilon	Aquacel	J & J Kling Bandage Rolls	
Systagenix	Kendall Amorphous	Aquacel AG	ADB Pads	
Mepitel	Sliverseal	Aquacel AQ Extra		
Restore	Prisma	Optilock		
Bridal Veil	Promogran Matrix	Repara Hydrocellular Foam		
Kendall Telfa				

**Table 3:** Comparison of pretreatment ulcer data between treatment groups*

	Ulcer size at baseline, cm^2^				
	Conv Care	Combined D-ADM	Combined GJ-ADM	Reyzelman et al^11^: GJ-ADM	Driver et al^12^: Integra
*n*	56	53	23	46	154
Mean	3.1	3.6	3.3	3.6	3.5
Median	2.2	1.9	2.2	2.2	…
SD	3.0	4.2	2.5	4.3	2.5
Range	0.8–14.2	0.8–21.8	1.0–10.5	0.6–23.3	…
*P* value vs Conv Care	…	.5764	.7827	…	…
*P* value vs GJ-ADM	…	.7819	…	…	…

*Conv Care indicates conventional care.

**Table 4:** Summary of results for DFU per protocol patients

	Conv Care	D-ADM (1 app)	GJ-ADM (1 app)	D-ADM (2 apps)	GJ-ADM (2 apps)	D-ADM (combined)	GJ-ADM (combined)
No. of patients at 12 wk	56	40	16	13	7	53	23
No. of patients at 16 wk	54	40	16	13	7	53	23
Mean time to complete wound closure, wk (*n*)	8.7 (26)	8.5 (33)	8.6 (11)	9.7 (3)	0 (0)	8.6 (36)	8.6 (11)
% of wounds completely closed by 12 wk (*n*)	41.1 (23)	65.0 (26)	56.3 (9)	15.4 (2)	0.0 (0)	52.8 (28)	39.1 (9)
		*P = .0203					
% of wounds completely closed by 16 wk (*n*)	48.1% (26)	82.5% (33)	68.8% (11)	23.1% (3)	0.0% (0)	67.9% (36)	47.8% (11)
		*P = .0003				*P = .0385	
Mean % reduction in wound area from baseline at 12 wk (*n*)	71.6 (56)	94.6 (40)	88.0 (16)	71.6 (13)	37.1% (7)	88.9 (53)	72.5 (23)
		*P = .0004	*P = .0410			*P = .0091	
Mean % reduction in wound area from baseline at 16 wk (*n*)	80.3 (54)	96.3 (40)	88.4 (16)	76.9 (13)	39.4 (7)	91.4 (53)	73.5 (23)
		*P = .0085					

*Statistical significance between the treatment group and the Conv Care group (*P* ≤ .05). Conv Care indicates conventional care.

**Table 5:** Comparison of percent wound reduction (baseline vs wound size) (%) for ulcers that did not completely heal at or on 16 weeks between treatment groups at 16-week follow-up*

	Conv Care	D-ADM (1 app)	GJ-ADM (1 app)	D-ADM (2 apps)	GJ-ADM (2 apps)	D-ADM (combined)	GJ-ADM (combined)
*n*	28	7	5	10	7	17	12
Mean	60.2	79.1	62.9	69.1	39.4	73.2	49.2
Median	86.4	83.0	61.5	73.7	72.2	81.8	68.8
SD	53.5	20.0	10.5	30.6	67.2	26.5	51.5
Range	−145.8 to 100.0	36.4–95.4	50.0–75.9	6.5–100.0	−66.7 to 97.1	6.5–100.0	−66.7 to 97.1
*P* value vs Conv Care	…	.1439	.8052	.5277	.4702	.2811	.5493
*P* value vs GJ-ADM	…	.1001	…	.3078	…	.1586	…

*Conv Care indicates conventional care.
